# Co-use of tobacco and cannabis: Complicated partnerships

**DOI:** 10.1192/j.eurpsy.2021.1534

**Published:** 2021-08-13

**Authors:** I. Ganhao, M. Trigo, A. Paixao

**Affiliations:** 1 Smoking Reduction And Cessation Programme, Centro Hospitalar Psiquiatrico de Lisboa, Lisbon, Portugal; 2 Psychology Unit, Centro Hospitalar Psiquiatrico de Lisboa, Lisbon, Portugal; 3 Clinic 4, Centro Hospitalar Psiquiatrico de Lisboa, Lisbon, Portugal

**Keywords:** tobacco smoking, cannabis, smoking cessation

## Abstract

**Introduction:**

Treating addiction is more challenging when there are co-addictions. Tobacco smoking is commonly associated with substance abuse, alcohol use disorders, excessive caffeine intake and pathological gambling among other addictions. Smoking reduction and cessation programmes´ objectives benefit from interventions targeting co-addictions.

**Objectives:**

Difficulties arising from smoking reduction and cessation in the context of co-use of cannabis prompt literature review and reflection of a smoking cessation programme team.

**Methods:**

Pubmed and Google Scholar literature search using terms smoking cessation / tobacco cessation and cannabis.

**Results:**

Co-use of tobacco and cannabis is: 1) very common, 2) associated with greater prevalence of morbidity and social problems, 3) associated with greater dependence of the other substances, 4) negatively influences quit outcomes of either, 5) increases the risk of relapse. Co-users are more likely to perceive the harmful effects of tobacco, have greater motivation and are more likely to quit tobacco than cannabis, which may be perceived as low risk. Treatment of either tobacco smoking or cannabis use may lead to compensatory increase in use of the other substance. There is a significant lack of literature on co-use treatment strategies.

**Conclusions:**

Co-use of tobacco and cannabis makes cessation and relapse prevention of either addiction more difficult and should be taken into account in smoking reduction and cessation programmes and in cannabis treatment interventions. Treatment targetting both tobacco and cannabis use, either simultaneously or sequentially, is likely more successful than interventions targeting only either one. Much remais to be studied on how to treat co-use of tobacco and cannabis.

